# An Unusual Case of Sinus of Valsalva Aneurysm in a GUCH Patient: An Unusual Side of the Aorto-Cardiac Fistula

**DOI:** 10.4021/cr27w

**Published:** 2011-07-25

**Authors:** Anna Balducci, Valentina Gesuete, Marianna Fabi, Fernando Maria Picchio, Gaetano Gargiulo

**Affiliations:** a Pediatric Cardiology Unit, S.Orsola-Malpighi Hospital, Bologna, Italy; b Pediatric Cardiac Surgery Unit, S.Orsola-Malpighi Hospital, Bologna, Italy

**Keywords:** Heart failure, Congenital heart disease, Tetralogy of Fallot

## Abstract

Sinus of Valsalva aneurysm can be congenital or acquired. The major complication is rupture: this can represent an evolution or an abrupt event. In case of rupture or of large aneurysm, a surgical approach is justified. We report a case of ruptured sinus of Valsalva aneurysm in a seventeen years old girl, who had underwent surgical correction of Fallot tetralogy. As soon as the rupture of sinus of Valsalva aneurysm is suspected, echocardiographic examination is the easiest and most straightforward tool to make a correct diagnosis.

## Introduction

Sinus of Valsalva aneurysms can be acquired or congenital, the latter being the most common type, with an incidence ranging from 0.1% to 3.5% of all congenital defects. Congenital aneurysms result from a disconnection between the aortic media and the fibrous anulus of the aortic valve, while others are the consequence of a connective tissue weakness caused by Marfan or Ehlers-Danlos syndromes [1]. Acquired sinus of Valsalva aneurysms can be the result of abrupt deceleration chest trauma, bacterial endocarditis, syphilis, tuberculosis, atherosclerosis, cystic medial necrosis [2, 3]. Rupture can produce an aorto-cardiac fistula and the diagnosis is really challenging because of the variability in presentation and clinical impact. The clinical manifestation depends on the cardiac chamber into the aorto-cardiac fistula occurs and on the time of onset of symptoms: it is often gradual but can also be abrupt and life-threatening. Moreover, symptoms can be due to coronary compression, therefore resulting in myocardial ischemia, or secondary to an obstruction of the right ventricular outflow tract. In reports the most commonly associated lesions are ventricular septal defects [4-6] and aortic valve regurgitation, but also tetralogy of Fallot, patent ductus arteriosus, coarctation of the aorta, bicuspid aortic valve [7, 8]. As soon as the condition is suspected transthoracic and transesophageal echocardiographic examination is the easiest and most straightforward tool to make a correct diagnosis. Pathological rupture of a sinus of Valsalva aneurysm most frequently involves the right sinus (90%), followed by the noncoronary (8%) and the left sinus (2%) [9]. The gold standard for treatment of ruptured sinus of Valsalva aneurysm is surgical repair by patch closure in order to avoid tension on the aortic anulus. The indication for repair of unruptured sinus of Valsalva aneurysm is controversial as the number of reports is limited,however an aggressive approach could be justified in case of complications such as right ventricular outflow tract obstruction, arrhythmias and for larger sinus of Valsalva aneurysms.

## Case Report

**Figure 1:**
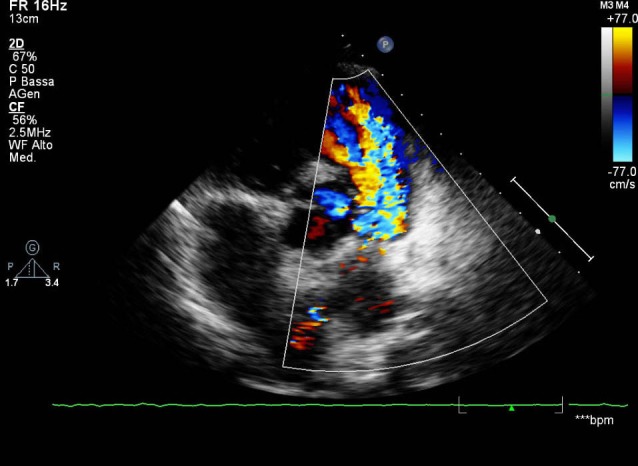
The transthoracic echocardiogram revealed an aneurysmatic left sinus of Valsalva, with a remarkable turbulent flow moving from the proximal aortic root to the right outflow tract.

We present the case of a seventeen years old girl complaining about shortness of breath under efforts occurring during the last months. She had repair of tetralogy of Fallot at the age of one year with trans-anular patching and proximal left pulmonary artery enlargement. An aneurysm of the pulmonary artery bifurcation at the site of the previous patch was discovered at routine magnetic resonance imaging (MRI) in January 2008 (33 x 35 mm). At that time, the patient was completely asymptomatic and the only other finding was a severe pulmonary insufficiency as a consequence of the surgical intervention. After the patient became symptomatic, a new MRI scan was undertaken showing an enlargement of the pulmonary aneurysm (50 x 42 mm) and an increase in right ventricle volume of about 50 ml/m^2^. At clinical examination, a new loud continuous widespread murmur was present and a thrill was easily palpable. Arterial pressure, cardiac frequency and oxygen saturation were within the normal range. Electrocardiogram didn’t differ from her previous one. The ultrasound scan revealed an aneurysmatic left sinus of Valsalva, with a remarkable turbulent flow moving from the proximal aortic root to the right outflow tract (Fig. 1). There were indirect signs of right ventricular volume overload and a diastolic reflow was detectable in the descending aorta, hence a fistulous communication between the aorta and the right ventricle was suspected. The diagnosis was confirmed by a transesophageal echocardiography and the girl underwent cardiac surgery. Aortotomy was performed in order to allow selective perfusion of the coronary ostia, then closure of the ruptured sinus and repair of the aneurysmatic pulmonary arterial tract were carried out successfully.

## Discussion

The cause of congenital sinus of Valsalva aneurysm is thought to be the incomplete fusion of the bulbar septum, which separates the aortic and pulmonary valves of the primitive bulbus cordis. This incomplete fusion would lead to weaker vascular wall therefore predisposing the high-pressure area of the sinuses to aneurysm formation. The rarity of congenital aneurysm from the left sinus of Valsalva could be due to the fact that the left cusp does not arise from the bulbar septum as the right and the noncoronary ones do [10]. Although the association between sinus of Valsalva aneurysm and tetralogy of Fallot is described, in this case we speculate that the left sinus could be congenitally predisposed to aneurysm formation. However the pulmonary arterial enlargement after cardiac surgery may also have played a role in its rupture in a “wearing and tearing fashion”. In addition, in tetralogy of Fallot the aortic root is slightly rotated thus allowing the rupture of the left sinus into the right ventricular outflow tract. Ultimately, both the congenital cardiac defect and the aneurysm of the pulmonary artery occurring after surgery generated the conditions for such an unusual and unpredictable case of rupture of the left sinus of Valsalva into the right outflow tract in an adult patient. Echocardiography was largely informative for the diagnosis. Actually, MRI failed to show clearly the rupture, but only demonstrated an enlargement of both right ventricle and pulmonary aneurysm. Angiography was not performed because we considered it a high risk procedure in this case and because the ultrasound scan provided all the information was needed.

## References

[1] Takach TJ, Reul GJ, Duncan JM, Cooley DA, Livesay JJ, Ott DA, Frazier OH (1999). Sinus of Valsalva aneurysm or fistula: management and outcome. Ann Thorac Surg.

[2] Harkness JR, Fitton TP, Barreiro CJ, Alejo D, Gott VL, Baumgartner WA, Yuh DD (2005). A 32-year experience with surgical repair of sinus of valsalva aneurysms. J Card Surg.

[3] Mayer JH, Holder TM, Canent RV (1975). Isolated, unruptured sinus of Valsalva aneurysm: serendipitous detection and correction. J Thorac Cardiovasc Surg.

[4] Mayer ED, Ruffmann K, Saggau W, Butzmann B, Bernhardt-Mayer K, Schatton N, Schmitz W (1986). Ruptured aneurysms of the sinus of Valsalva. Ann Thorac Surg.

[5] Vural KM, Sener E, Tasdemir O, Bayazit K (2001). Approach to sinus of Valsalva aneurysms: a review of 53 cases. Eur J Cardiothorac Surg.

[6] Kirali K, Guler M, Daglar B, Yakut N, Mansuroglu D, Balkanay M, Berki T (1999). Surgical repair in ruptured congenital sinus of Valsalva aneurysms: a 13-year experience. J Heart Valve Dis.

[7] Sundeen JT, Bloom S (1987). Sinus of Valsalva aneurysm associated with multiple conotruncal congenital malformations. Hum Pathol.

[8] Azakie A, David TE, Peniston CM, Rao V, Williams WG (2000). Ruptured sinus of valsalva aneurysm: early recurrence and fate of the aortic valve. Ann Thorac Surg.

[9] Dev V, Goswami KC, Shrivastava S, Bahl VK, Saxena A (1993). Echocardiographic diagnosis of aneurysm of the sinus of Valsalva. Am Heart J.

[10] Jones AM, Langley FA (1949). Aortic sinus aneurysms. Br Heart J.

